# The association between season and meteorological factors on clinical outcomes after fresh embryo transfer: a cohort study from Central Plains, China

**DOI:** 10.3389/fendo.2025.1486633

**Published:** 2025-05-21

**Authors:** Hao Li, Xin Li, JinYu Li, YuMeng Ma, Gang Li

**Affiliations:** Reproductive Medical Center, First Affiliated Hospital of Zhengzhou University, Zhengzhou, Henan, China

**Keywords:** art, season, temperature, live birth rate, clinical pregnancy rate

## Abstract

**Objective:**

To investigate whether seasonal variations and meteorological factors influence pregnancy outcomes in women undergoing *in vitro* fertilization/Intracytoplasmic sperm injection and embryo transfer(IVF/ICSI-ET) treatment.

**Materials and methods:**

We conducted a retrospective cohort study of women who underwent IVF/ICIS-ET for the first time at the Reproductive Medicine Center of the First Affiliated Hospital of Zhengzhou University from January 1, 2011, to December 1, 2021. A total of 24420 cycles were collected. They were divided into four groups according to the oocyte retrieval date. The main outcome measures were clinical pregnancy rate and live birth rate. Binary logistic regression was used to explore the factors affecting clinical pregnancy rate and live birth rate.

**Results:**

In this study, the live birth rate of cycles with oocyte retrieval performed in summer was significantly higher than those in spring, autumn, and winter (61.24% [4859/7934] vs. 59.09% [3074/5202] vs. 58.89% [3676/6242] vs. 57.70% [2909/5042]; *P*<0.001), with the summer group also exhibiting the lowest abortion rate among the four seasons. Notably, despite these differences, no statistically significant variations were observed in biochemical pregnancy rates or clinical pregnancy rates across the groups (*P*>0.05). Taking spring as the reference, the live birth rate in summer was higher (aOR=1.079,95% CI: 1.004-1.160), and the abortion rate was lower (aOR = 0.841,95% CI: 0.746-0.948). The live birth rate and abortion rate in autumn and winter were not significantly different from spring. Multivariate logistic regression analysis showed higher daily average temperature, and humidity at the time of oocyte retrieval increased the live birth rate (OR = 1.005, 95% CI = 1.002–1.007; OR = 1.004, 95% CI = 1.001–1.006).

**Conclusions:**

In women who undergo IVF/ICSI treatment, the season ambient temperature, and humidity on the date of the oocyte retrieval may impact embryo development and live birth.

## Introduction

Assisted reproductive technology (ART) is a medical technique used to treat infertility, including *in vitro* fertilization (IVF), intracytoplasmic sperm injection (ICSI), and embryo transfer (ET) The success rate of ART is affected by many factors, including the patient’s age, health status, reproductive system condition and treatment plan. The observed seasonality of natural human pregnancy and birth rates is widely recognized by epidemiologists (1). This phenomenon raises the question of whether ART also exhibits seasonal patterns—a subject that has garnered interest within the research community. Fluctuations in annual ART pregnancy rates have been noted by many experts, presenting variations that are challenging to ascribe merely to technological advancements or laboratory culture conditions. Consequently, the focus has shifted to extrinsic factors, including seasonal shifts and light exposure, which may influence these rates. Some studies deny any correlation between the seasons and reproductive outcomes (2–4), In contrast, other research reports a definitive seasonal influence (5–7), with some attributing it to temperature changes (6), and others pointing to the variation in daylight hours (7). Such disparate findings could stem from differences in research design, geographical settings, ethnic backgrounds, climate conditions, and protocols for ovulation induction. To provide clarity on this matter, our study conducts a retrospective analysis of the relationship between clinical pregnancy outcomes and meteorological factors among patients undergoing ART at our center. Spanning 11 years, this investigation seeks to discern if there is indeed a connection between the meteorological environment of Central China and the success rates of assisted reproduction in this locale.

## Materials and methods

### Data source

The clinical records of patients who received assisted reproductive treatment at the Reproductive Center of the First Affiliated Hospital of Zhengzhou University from January 2011 to December 2021 were collated for this retrospective study. Inclusion criteria (1): Couples who underwent *in vitro* fertilization-embryo transfer (IVF-ET) or intracytoplasmic sperm injection-embryo transfer (ICSI-ET) within the first cycle (2); Couples who successfully underwent oocyte retrieval, embryo formation, and fresh transfer after controlled ovarian stimulation (COS) (3); Men and women with comprehensive body mass index (BMI) data and documented follow-ups. Exclusion criteria include (1): Sperm donation, oocyte donation, or thawing cycles (2); Pre-implantation genetic diagnosis/screening (PGD/PGS) cycles (3); Intrauterine insemination cycles (4); Cycles with incomplete follow-up data (5); Women and/or men with chromosomal abnormalities and/or genetic diseases.

### Climate data

The meteorological information for Zhengzhou was obtained from the Resources and Environment section of the China Statistical Yearbook, with a focus on the monthly average temperature, humidity, and total sunlight hours. Grouping by Meteorological Seasons: For a more accurate reflection of the climate’s influence, patients were grouped based on the date of oocyte retrieval corresponding to meteorological seasons: spring (March to May), summer (June to August), autumn (September to November), and winter (December to February of the subsequent year).Climatic Factors Analysis: To assess the impact of climate on reproductive outcomes, the monthly temperature, humidity, and cumulative sunlight exposure were categorized into specific groups. Temperature was divided into low (<10°C), medium (10-20°C), and high (>20°C) temperature groups. Humidity was categorized using tertiles into low (<53%), medium (53-61%), and high (>61%) humidity groups. Cumulative sunlight was segmented into low (≤133.2h), medium (134.8-172h), and high (≥172.3h) sunlight groups.

### IVF protocol

Our research employed controlled ovarian stimulation cycles using diverse strategies, such as long luteal phase, long follicular phase, and antagonist protocols. The selection of these protocols was primarily based on individual factors like women’s serum hormone levels, age, and ovarian functionality. This tailored approach guided the administration of ovulation induction treatments, ensuring that drug dosages were adjusted in accordance with each patient’s specific circumstances throughout the treatment period. In the downregulation stage, at the onset of the menstrual cycle (day 2–3), we administer 3.75 mg of a long-acting gonadotropin-releasing hormone (GnRH) agonist (Diphereline, Beaufour Ipsen, France) via subcutaneous injection to achieve pituitary downregulation. Following a period of 30–42 days, we conduct a vaginal ultrasound and measure serum levels of follicle-stimulating hormone (FSH), luteinizing hormone (LH), estrogen (E2), and progesterone (P). This assessment ensures that patients meet the downregulation criteria, characterized by the absence of functional cysts larger than 10 mm in diameter, FSH levels below 5 IU/L, and LH levels below 3 IU/L. Once these standards are met, controlled ovarian hyperstimulation is initiated. For ovulation induction and oocyte retrieval, the gonadotropin (Gn) dosage (GONAL-f, Merck Serono, Germany) is tailored to each patient’s age, anti-Müllerian hormone (AMH) level, antral follicle count (AFC), BMI, and baseline serum FSH levels. Adjustments to the Gn dosage and the inclusion of human menopausal gonadotropin (LeBold, Zhuhai Livzon Pharmaceutical, China) depend on follicle size and hormone levels. Ovulation is triggered when one dominant follicle reaches at least 20 mm and a minimum of three dominant follicles are 17 mm or more in diameter. For this purpose, we use 250 mg of Azer (Merck Serono, Italy) combined with 2000 IU of human chorionic gonadotropin (hCG) (Zhuhai Livzon Pharmaceutical, China). Oocyte retrieval is then performed under vaginal ultrasound guidance, 36–37 hours after administering the trigger injection.

### Embryo culture and transfer

The follicles were retrieved under ultrasound guidance 36–37 hours after triggering. On the same day, semen specimens were collected from male partners via masturbation following standardized protocols. All study participants adhered to a prescribed sexual abstinence period of 2 to 7 days prior to semen collection, following established protocols. IVF and ICSI inseminations were both done at 38–40 h after triggering. Embryos were then cultured in a continuous medium in an incubator maintained at 37°C with a CO2 concentration of 6%. And insemination was assessed on the first day (the day after insemination), and the quality of embryos at the Cleavage stage was assessed on the third day. Fertilization was assessed on the first day following oocyte retrieval, with the presence of a double pronucleus (2PN) indicating normal zygotes. These zygotes were then transferred to cleavage fluid for further culture. On the third day, we evaluated cleavage and development, assessing embryo quality based on blastomere number, diameter, morphology, and developmental rate. Fresh embryo transfer was conducted based on embryo quality, endometrial condition, and patient factors.

### Outcome measures

The primary outcomes were clinical pregnancy and live birth, while secondary outcomes included biochemical pregnancy and early miscarriage. Clinical pregnancy was confirmed by ultrasonographic visualization of one or more gestational sacs or definitive clinical signs. Live birth was defined as the birth of a fetus that showed any signs of life post-expulsion or extraction, regardless of gestational age. Biochemical pregnancy refers to a pregnancy identified solely through hCG detection in serum or urine without clinical development. Early miscarriage was characterized as the spontaneous loss of a clinical pregnancy before 12 completed gestational weeks. Evaluation measures included: 1) ovarian response indicators: such as total and duration of Gn usage, and number of oocytes retrieved; 2) embryo quality indicators: including the number and rate of 2PN fertilizations; and 3) pregnancy outcomes: rates of biochemical pregnancy, clinical pregnancy, early miscarriage, and live birth.

### Statistical analysis

Statistical analysis was performed using SPSS 26.0 software. Continuous variables were expressed as mean ± standard deviation and categorical variables as frequency and percentage. We compared baseline characteristics across four groups. For continuous variables, one-way ANOVA or Kruskal-Wallis tests were used for homogeneous variance groups, while the LSD-t test was utilized for within-group pairwise comparisons. In cases of unequal variances, the Games-Howell test was applied. Multivariable logistic regression analysis helped assess the BMI-outcome relationship for dichotomous results, with odds ratios (ORs) and 95% confidence intervals (CI). A p-value of <0.05 was considered statistically significant (two-sided).

## Result

### Clinical characteristics of four groups of patients according to the season of oocyte retrieval date

**Table 1** describes the clinical character of patients in 24,420 IVF/ICSI treatment cycles corresponding to different seasons, all of which met the study’s inclusion criteria. The number of cycles varies across different seasons, with the highest count in summer (7934) and the lowest in winter (5042). There is no significant difference in female age among different seasonal groups, while the male age in the winter group is slightly higher than that in other seasonal groups (P=0.010). The female BMI in the spring group is the highest and significantly higher than that in the summer and autumn groups (P < 0.010), but there is no statistical difference in male BMI. However, summer stands out as the season with the lowest baseline FSH levels and the highest Antral Follicle Counts (AFC) among patients, a notable distinction supported by statistical significance (P<0.05). Furthermore, our study identified a clear seasonal pattern in sperm quality: both total sperm count and progressive motility displayed the lowest values in the autumn group and peaked in the spring group, with autumn also recording the highest number of immotile sperm (P<0.05). This seasonality may be attributed to the approximately 74-day cycle of spermatogenesis, with adverse effects of high summer temperatures on sperm quality becoming evident in the autumn, whereas the optimal conditions of winter contribute to better sperm quality observed in the spring.\

**Table 1:** Clinical characteristics of four groups of patients according to the season of oocyte retrieval date.

Parameter	Spring Group	Summer Group	Autumn Group	Winter Group	*P*
Cycle	5202	7934	6242	5042	
Female Age (year)	30.44 ± 4.56	30.40 ± 4.50	30.44 ± 4.39	30.57 ± 4.56	0.214
Male Age (year)	31.64 ± 5.30	31.45 ± 5.27^c^	31.51 ± 5.04^c^	31.75 ± 5.43	0.01
Female BMI (kg/m^2^)	22.86 ± 3.14^bc^	22.80 ± 3.24^b^	22.59 ± 3.17^c^	22.71 ± 3.06	0.001
Male BMI (kg/m^2^)	24.96 ± 3.47	25.04 ± 3.58	24.97 ± 3.64	25.08 ± 3.56	0.224
Basal FSH (IU/L)	6.98 ± 2.32^b^	6.97 ± 2.37^b^	7.08 ± 2.38	7.00 ± 2.25	0.017
AFC	12.97 ± 6.26^abc^	13.53 ± 6.47^c^	13.41 ± 6.49	13.24 ± 6.42	0.001
Total sperm count*10^6^	182.89 ± 2.71	173.91 ± 2.07	160.98 ± 2.39	172.65 ± 2.84	0.001
PRM	27.85 ± 26.97^abc^	25.60 ± 27.49^bc^	22.60 ± 26.59^c^	23.30 ± 26.34	0.001
NRP	14.41 ± 10.01^b^	14.44 ± 10.49^b^	13.58 ± 10.27^c^	14.17 ± 10.35	0.001
I’M	37.76 ± 25.74^b^	37.02 ± 25.91^bc^	39.71 ± 26.80	38.87 ± 26.08	0.001

Data are presented as mean ± S.D. a indicates P<0.05, compared with the summer group; b indicates P<0.05, compared with the autumn group; c indicates P<0.05, compared with the winter group. PRM, progressive motility; NPR, non-progressive motility; IM, immotility.

### Assisted pregnancy outcomes of four groups according to the season of oocyte retrieval date.

**Figure 1:**
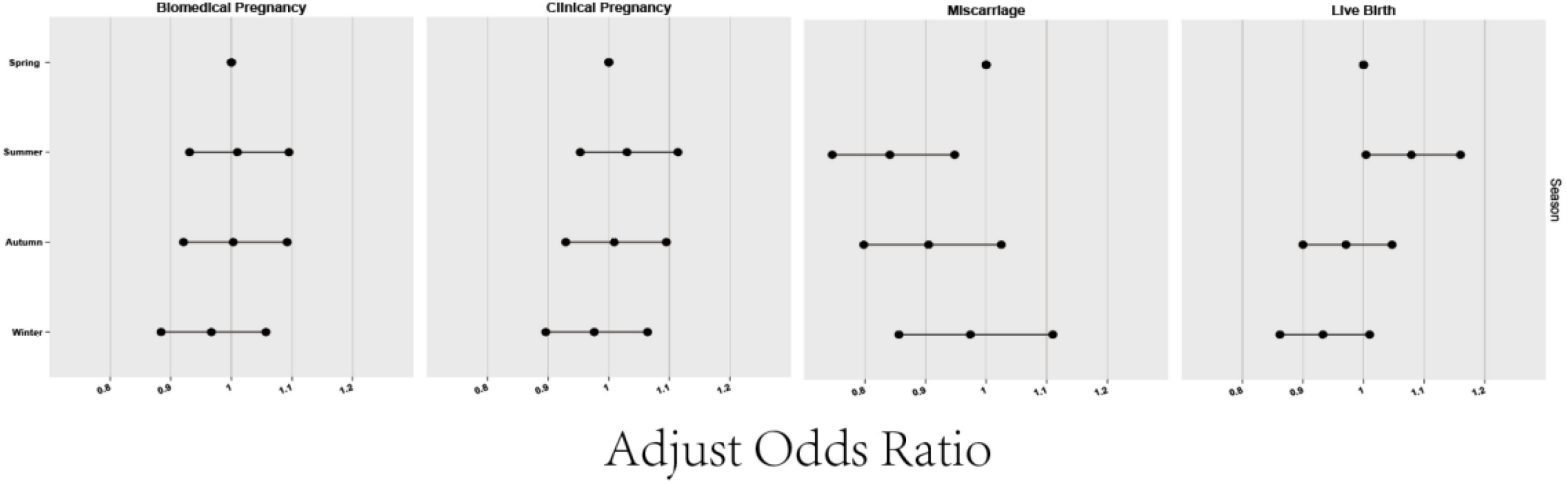
Relation between the season of oocyte retrieval date and pregnancy outcomes.

**Figure 1** and **Table 2** illustrates the laboratory and clinical data of patients grouped by season. Spring emerged as the season with the shortest gonadotropin (GN) administration duration for oocyte retrieval and the lowest follicle-stimulating hormone (FSH) usage (p<0.05). However, this group also had the thinnest endometrial lining post-ovulation induction, though not significantly thinner (p>0.05). In regards to oocyte retrieval, despite patients in summer recording the highest antral follicle count (AFC), the actual yield was the lowest. In contrast, the autumn group demonstrated the highest oocyte retrieval count compared to other seasons (*P*<0.05). The data did not reveal significant variances in IVF fertilization rates, IVF-2PN (pronuclei) rates, or ICSI-2PN rates among the seasonal groups (*P*>0.05). This indicates that the primary factor affecting the quantity of IVF and ICSI fertilized oocytes and 2PN rates was the number of oocytes retrieved, rather than seasonal differences. The comparison of biochemical and clinical pregnancy rates across all groups showed no statistically significant discrepancies (all *P*>0.05). However, miscarriage and live birth rates manifested notable seasonal differences (*P*<0.01 for miscarriage rates; *P*<0.001 for live birth rates). Detailed analysis pointed out higher miscarriage rates in the spring [15.42% (572/3708)] and winter [15.16% (543/3581)] when compared to the summer [13.19% (754/5718), *P*<0.01]. No conspicuous differences were noted in miscarriage rates between other groups. Live birth rates in the spring [59.09% (3074/5202)], autumn [58.89% (3676/6242)], and winter [57.70% (2909/5042)] proved to be inferior to those in the summer [61.24% (4859/7934), *P*<0.001], while comparisons among the remaining groups again showed no significant differences.

**Table 2:** Assisted pregnancy outcomes of four groups according to the season of oocyte retrieval date.

Parameter	Spring Group	Summer Group	Autumn Group	Winter Group	*P*
Gn day	12.00 ± 2.19^abc^	12.51 ± 2.25	12.52 ± 2.24	12.53 ± 2.27	0.001
The total dose of FSH	1968.91 ± 724.61 ^abc^	1997.77 ± 736.32^b^	2043.23 ± 784.05^c^	2012.50 ± 732.79	0.001
Endometrial thickness(mm)	12.25 ± 3.62	12.26 ± 3.23	12.25 ± 3.16	12.32 ± 4.27	0.752
No. of retrieved oocytes	12.51 ± 5.82 ^b^	12.46 ± 5.70 ^b^	12.73 ± 5.96	12.59 ± 5.89	0.048
Number of fertilized oocytes	12.07 ± 5.77 ^b^	11.96 ± 5.79 ^bc^	12.34 ± 5.95	12.25 ± 5.94	0.005
IVF Fertilization rate(%)	94.63 ± 18.63	93.45 ± 20.62	92.51 ± 22.13	93.61 ± 20.08	0.638
IVF- No. of 2PN	7.98 ± 4.31	7.88 ± 4.30 ^b^	8.09 ± 4.40	8.01 ± 4.40	0.086
IVF-2PN rate(%)	71.10 ± 19.96	71.69 ± 21.33	72.90 ± 19.47	78.28 ± 90.13	0.23
ICSI- Number of fertilized oocytes	11.94 ± 5.50	12.29 ± 5.37	12.35 ± 5.72	12.01 ± 5.58	0.142
ICSI- Fertilization rate(%)	84.17 ± 16.37	82.04 ± 16.70	78.84 ± 19.43	83.40 ± 17.23	0.18
ICSI- No. of 2PN	8.09 ± 4.13	8.12 ± 4.07	8.21 ± 4.24	7.98 ± 4.14	0.572
ICSI-2PN rate(%)	72.86 ± 16.83	80.87 ± 16.02	69.14 ± 18.39	70.89 ± 18.17	0.063
Biomedical pregnancy, rate(%)	74.47 ± 0.44	74.96 ± 0.43	74.99 ± 0.43	74.49 ± 0.44	0.659
Clinical pregnancy rate (%)	71.16 ± 0.45	72.03 ± 0.45	71.74 ± 0.45	70.90 ± 0.45	0.489
Miscarriage rate (%)	15.45 ± 0.36^a^	13.20 ± 0.34^c^	14.00 ± 0.35	14.64 ± 0.36	0.007
Live birth rate(%)	58.80 ± 0.49 ^a^	61.38 ± 0.49^bc^	58.57 ± 0.49	58.15 ± 0.49	0.001

Data are presented as mean ± S.D. a indicates *P*<0.05, compared with the summer group; b indicates *P*<0.05, compared with the autumn group; c indicates *P*<0.05, compared with the winter group.

### The multivariate logistic regression analysis of season and pregnancy outcomes of the four groups

**Table 3** elaborates on the impact of the oocyte retrieval season on pregnancy outcomes, employing binary logistic regression to control for confounders. The study considered variables such as biochemical pregnancy, clinical pregnancy, miscarriage, and live birth, adjusted for the age of both female and male partners, baseline AFC, motile sperm count, and the season of oocyte retrieval (categorized as spring, summer, autumn, and winter, with spring serving as the reference group). Our findings indicate that the summer group exhibited a significantly higher rate of miscarriages compared to the spring group (aOR=0.841, 95% CI: 0.746-0.948). On the other hand, the miscarriage rates for both the autumn group (aOR=0.905, 95% CI: 0.798-1.025) and winter group (aOR=0.974, 95% CI: 0.856-1.110) were not statistically different from those in the spring group, pointing to a degree of seasonality in reproductive outcomes. Analysis of live birth rates revealed that the summer group had a statistically significant higher rate compared to the spring group (aOR=1.079, 95% CI: 1.004-1.160, *P*<0.05), suggesting a noteworthy seasonal influence. Conversely, the autumn (aOR=0.971, 95% CI: 0.900-1.047) and winter (aOR=0.933, 95% CI: 0.862-1.010) groups did not demonstrate a statistically significant difference in live birth rates when compared to the spring, indicating a more complex interaction between seasonality and reproductive outcomes. The seasonality of oocyte retrieval appears to notably affect both miscarriage and live birth rates.

**Table 3:** The multivariate logistic regression analysis of season and pregnancy outcomes of the four groups.

Parameter	Spring	Summer	Autumn	Winter
Biomedical pregnancy rate	Reference	1.010 (0.931-1.095)	1.003 (0.921-1.092)	0.967 (0.884-1.057)
Clinical pregnancy rate		1.030 (0.953-1.114)	1.009 (0.929-1.095)	0.976 (0.896-1.064)
miscarriage rate		0.841 (0.746-0.948)	0.905 (0.798-1.025)	0.974 (0.856-1.110)
Live birth rate		1.079 (1.004-1.160)	0.971 (0.900-1.047)	0.933 (0.862-1.010)

Data were expressed as OR (95%CI). All models are adjusted according to female age, male age, AFC, and PNR.

### The multivariate logistic regression analysis of individual meteorological factors and pregnancy outcomes

**Figure 2:**
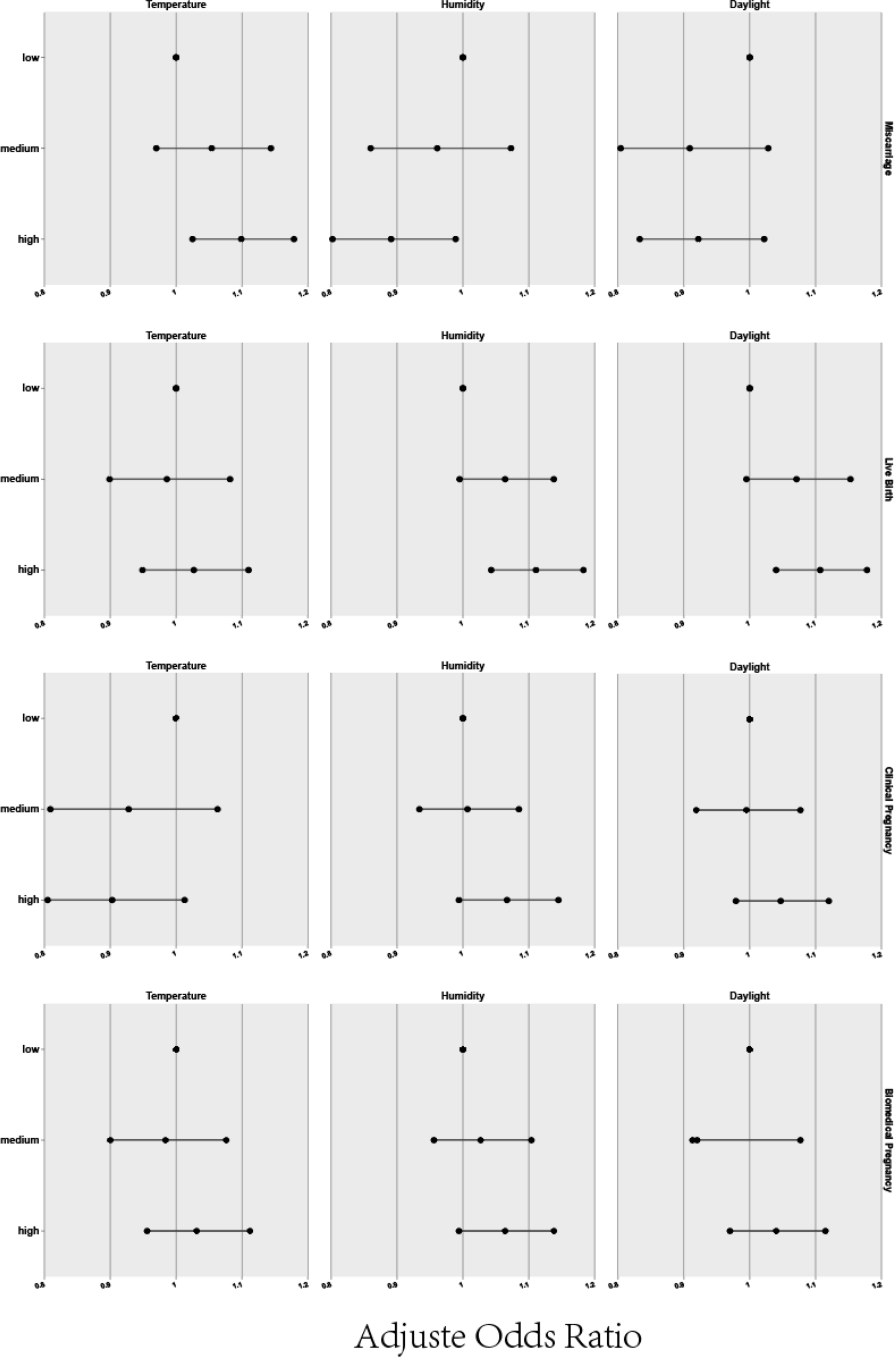
Relation between individual meteorological factors and pregnancy outcomes.

Beyond the raw delineation of seasons, we delved into various environmental factors such as temperature, humidity, and daylight duration, which inherently differ across seasons, to discern their potential effects on pregnancy outcomes. For this purpose, climate factors were categorized into three groups to elucidate their individual and combined impact. **Figure 2** and **Table 4** presents a detailed analysis of the relationship between individual meteorological factors and pregnancy outcomes, following adjustments for potential confounders using binary logistic regression. The study specifically considered observation indicators such as biochemical pregnancy, clinical pregnancy, miscarriage, and live birth, while controlling for factors including the female and male age, baseline AFC, motile sperm count, along with meteorological factors associated with oocyte retrieval (temperature, humidity, daylight), all compared to a reference lower data group Regression analysis showed a positive correlation between higher temperatures and a greater live birth rate when compared to cooler conditions (OR=1.099, 95% CI: 1.025-1.179), while increased humidity was associated with both reduced miscarriage rates and higher live birth rates (miscarriage rate OR=1.099, 95% CI: 1.025-1.179; live birth rate OR=1.111, 95% CI: 1.043-1.183) as opposed to lower humidity levels. Furthermore, longer daylight hours also corresponded with improved live birth rates vis-a-vis shorter daylight durations (OR=1.107, 95% CI: 1.040-1.178).

**Table 4:** The multivariate logistic regression analysis of individual meteorological factors and pregnancy outcomes.

Parameter	temperature			humidity			daylight		
	low	medium	high	low	medium	high	low	medium	high
Biomedical pregnancy rate	Reference	0.986(0.899-1.082)	1.027(0.949-1.110)	Reference	1.007(0.934-1.085)	1.067(0.994-1.145)	Reference	0.92(0.913-1.077)	1.040(0.970-1.115)
Clinical pregnancy rate		0.984(0.900-1.076)	1.031(0.956-1.112)		1.027(0.956-1.104)	1.064(0.994-1.138)		0.995(0.919-1.077)	1.047(0.979-1.120)
Miscarriage rate		0.928(0.809-1.063)	0.903(0.805-1.013)		0.961(0.860-1.073)	0.891(0.802-0.989)		0.909(0.804-1.028)	0.922(0.833-1.022)
Live birth rate		1.054(0.970-1.144)	1.099(1.025-1.179)		1.064(0.995-1.138)	1.111(1.043-1.183)		1.071(0.995-1.153)	1.107(1.040-1.178)

Data were expressed as OR (95%CI). All models are adjusted according to female age, male age, AFC, and PNR.

**Table 5** shows that when assessing temperature, humidity, and daylight duration as continuous variables, our analysis suggests that for each 1°C temperature increase, the live birth rate could improve by 0.5%, and each 1% increase in humidity potentially increases the biochemical pregnancy rate by 0.3% and the live birth rate by 0.4%. However, variations in daylight duration did not yield a statistically significant effect on pregnancy outcomes.

**Table 5:** The relationship between individual meteorological factors(continuous variable) and pregnancy outcomes.

parameter	temperature		humidity		daylight	
	OR 95%CI	P	OR 95%CI	P	OR 95%CI	P
Biomedical pregnancy rate	1.002 (0.999-1.005)	0.239	1.003 (1.000-1.005)	0.037	1.000 (1.000-1.001)	0.304
Clinical pregnancy rate	1.002 (0.999-1.005)	0.201	1.002 (1.000-1.005)	0.059	1.000 (1.000-1.001)	0.39
Miscarriage rate	0.996 (0.992-1.000)	0.073	0.997 (0.993-1.001)	0.148	1.000 (0.999-1.001)	0.645
Live birth rate	1.005 (1.002-1.007)	0.001	1.004 (1.001-1.006)	0.002	1.001 (1.000-1.001)	0.057

Data were expressed as OR (95%CI). All models are adjusted according to female age, male age, AFC, and PNR.

## Discussion

Extensive research has explored the myriad influences on pregnancy outcomes, yet the underlying mechanisms frequently remain elusive. Factors traditionally held in high regard include the woman’s age, embryo quality, and endometrial receptivity—especially during the critical window of embryo implantation. In this study, we compared live birth rates as the primary pregnancy outcome, live birth is the most immediate outcome that patients care about, and it is also the ultimate indicator for evaluating the effectiveness of assisted reproductive technologies such as *in vitro* fertilization (IVF). In ART, the literature presents a dichotomy, with some domestic and international studies suggesting possible seasonal effects, while others dispute the existence of such trends, leading to mixed conclusions. Ambiguities in existing research—stemming from diverse ovarian stimulation protocols, varying criteria for patient inclusion based on season, and different climatic backgrounds—make it challenging to directly compare findings across studies. Moreover, prior investigations often overlook the specific elements of seasonality, such as temperature, humidity, and daylight duration.

Our investigation probed the association between pregnancy outcomes after fresh embryo transfer and multiple parameters, including the season of oocyte retrieval, average monthly temperature, humidity, and sunlight exposure on the day of oocyte retrieval. The study concludes that temperature, humidity, and sunlight rank as significant meteorological factors influencing pregnancy outcomes. Notably, seasons with higher average temperatures, humidity, and sunlight exposure—typically summer—show an enhanced rate of pregnancy compared to seasons with generally lower levels of these variables, such as winter. Meteorological elements such as temperature, humidity, and sunlight were dissected to reveal an association with reproductive outcomes. Higher monthly averages of these components correlated with increased live birth rates. Further analysis, treating these factors as continuous variables, pinpointed that upticks in temperatures and humidity are linked with a rise in live birth rates. This gradient relationship underscores the independent effects of summer’s intensified warmth, humidity, and sunlight on live births. Consequently, the synergy of these three factors in summer could explain the season’s particularly high live birth rates. Parallel findings emerged from Wood and colleagues’ study on IVF and ICSI outcomes in the UK, which indicated that summer surpasses winter in fostering the optimal conditions for pregnancy (8).

The fertility implications of seasonal changes are two-fold: weather conditions and temperature play a crucial role in male sperm quality (9) while sunlight exposure is a determinant of female ovulation patterns. Of note is the germinal epithelium in the testes, which is acutely temperature-sensitive, making it vulnerable to even slight increments in heat that can inhibit sperm production. For optimal spermatogenesis, the testes must maintain a temperature of 2-6°C below the body’s core temperature. Deviations from this thermal niche can be detrimental to spermatogenesis, thereby influencing conception rates (10, 11)., High temperatures have been shown to negatively impact male fertility by inhibiting sperm formation and hormone synthesis, resulting in decreased sperm count and compromised quality in ejaculation. This association is supported by existing research indicating that sperm concentration and rapid motility typically peak in spring, with a notable decline in autumn—an observation that is consistent with our own findings (12). From a practical standpoint, the seasonal variation in semen quality may seem to pose a challenge for IVF/ICSI outcomes. However, the IVF/ICSI process involves rigorous semen preparation to isolate the most viable sperm for fertilization, rendering the seasonal influence less significant. Even in cases where viable sperm quantities are adversely affected by the season, ICSI can still provide a successful fertilization rate by focusing on selecting individual sperm. It is also apparent that the ambient temperature at the time of oocyte retrieval is an important factor affecting IVF/ICSI success rates during different seasons. Despite the observed relationship, the influence of ambient temperatures on ovarian function and its implications for reproductive technology remains an area for further investigation. A study assessing temperature’s impact on ovarian function revealed a negative correlation between high temperatures and AFCs among infertile patients, suggesting a decrease in ovarian reserve (13). Contrary to prior evidence, our data demonstrate that AFCs during summer oocyte retrieval cycles were significantly elevated compared to spring, autumn, and winter cycles, a finding that conflicts with established literature on seasonal influences in ovarian response. Although AFC is a marker of ovarian reserve, it may not directly reflect oocyte quality. Interestingly, there could be a positive relationship between higher ambient temperatures and both increased AFCs and potentially improved oocyte quality. Moreover, our study’s timelines differ from previous investigations. Gaskins. Et (13) measured body temperature 90 days prior to testing the ovarian reserve, coinciding with follicle recruitment phases. In contrast, our temperature measurements were concurrent with oocyte extraction, providing a current snapshot of conditions during the crucial 36-hour window of oocyte maturation. Thus, our data suggest that higher ambient temperatures might influence oocyte maturation and quality more directly.

Melatonin (14), a hormone with antioxidant properties has been shown to interact with the reproductive system. Secreted by the pineal gland, melatonin’s production is higher during periods of darkness, regulating seasonal reproductive changes by controlling gonadotropin-releasing hormone (GnRH) secretion. Seasonal variations in melatonin could lead to changes in neurotransmitter release and influence the plasticity of GnRH neurons. In humans, melatonin concentrations in follicular fluid before ovulation are higher than in serum, and these levels align with seasonal light-dark variations. This indicates melatonin’s potential role in sex hormone production and its impact on oocyte maturation and quality. Granulosa cells within mature follicles are known to secrete melatonin. For individuals with Polycystic Ovary Syndrome (PCOS), who may require oocyte *in vitro* maturation (IVM), supplementing the IVM culture medium with melatonin has been found to enhance cytoplasmic maturation and clinical outcomes (15) A study involving low-quality oocytes demonstrated that the addition of melatonin, inositol, and folic acid to the treatment protocol significantly improved oocyte quality and subsequent pregnancy success rates (16). The majority of participants in these studies underwent pituitary down-regulation with GnRH analogs and received exogenous gonadotropins for ovarian stimulation. This treatment approach suggests that any direct influence of light exposure on the participants may be mitigated due to the induced suppression of GnRH neuron activity. Moreover, melatonin receptors are widely distributed in peripheral tissues, suggesting a potentially more immediate regulatory role of melatonin outside the central nervous system. Studies indicate that the length of daylight (photoperiod) affects melatonin levels, subsequently influencing steroid hormone secretion. This may have a consequential effect on the endometrium’s receptivity, further impacting reproductive outcomes.

Human 7-dehydrocholesterol can be transformed into vitamin D through exposure to ultraviolet rays, with summer typically yielding higher vitamin D levels in the serum due to increased sunlight. The relationship between vitamin D levels and *in vitro* fertilization (IVF) success rates, however, remains a subject of debate. While some studies associate elevated vitamin D with a greater likelihood of IVF success (17–20) others do not support this correlation (21–23). A systematic review and meta-analysis incorporating findings from 11 cohort studies reported a positive relationship between higher vitamin D concentrations and improved chances of clinical pregnancy and live birth in women utilizing assisted reproductive technologies (24).

Despite these findings, the inconsistency among studies means that the individual contributions of vitamin D and melatonin to folliculogenesis are yet to be definitively determined. As a result, the current body of evidence does not allow for definitive claims regarding a causal link between these substances and the quality of oocytes in response to ovarian stimulation. To date, no studies have examined the effects of ambient temperature and humidity on serum melatonin and vitamin D levels. Moreover, sunlight’s role in IVF treatments may involve other elements such as vitamin D3, serotonin, dopamine, and opioids (17, 25–27). These insights propose that seasonal changes could indirectly affect pregnancy outcomes, not only by potentially impacting the quality of sperm and oocytes but also through other pathways. Our research posits that the summer season, characterized by prolonged sunlight exposure and likely increased vitamin D levels, is associated with better pregnancy outcomes post-IVF/ICSI treatment. While the detailed mechanisms by which seasons affect the outcomes of assisted reproductive pregnancy cycles remain elusive, the concept of photoperiodism in humans is evident. The duration of sunlight seems to forge a connection between the seasons and enhanced clinical success in fresh embryo transfers. Indeed, seasonal variables, including climate and environmental factors, likely play a role in IVF treatment results.

Air quality has shown a significant correlation with reproductive outcomes, with adverse effects being observed in both natural and assisted conceptions. Particulate matter, especially Particulate Matter(PM)10 (28) and PM2.5 (29), is associated with decreased fertility, an increased risk of infertility, lower live birth rates, and higher miscarriage rates among IVF patients. Elevated nitrogen dioxide (NO_2_) exposure correlates with increased miscarriage rates in the general population and reduced live birth rates in those undergoing IVF. Additionally, sulfur dioxide (SO_2_) exposure is linked to *in vitro* DNA damage and a rise in miscarriage occurrences. Research from a regional institution underscores that atmospheric levels of PM10 and SO_2_ have tangible effects on pregnancy outcomes (30).

Our study’s extensive dataset, analyzing over 24,000 ART cycles and encompassing a broad range of ovulation induction protocols and infertility causes, ensured a robust sample size across most BMI categories, enhancing the relevance and generalizability of our findings. All cycle data were sourced from a single large institution, which helped maintain consistency in physician practices, treatment plans, and patient profiles. We focused on the first fresh ART cycle for each patient to reduce confounding factors from repeated or frozen cycles, thereby providing a more precise estimation of outcomes. However, our study is not without limitations. Firstly, the major limitation of this study is its retrospective nature. Secondly, we collected retrospective data from a single center, the First Affiliated Hospital of Zhengzhou University. Therefore, the research findings may be difficult to apply to other regions with different climates. In addition, indoor heating is quite common in some areas of Henan Province, which may have influenced the research results. Similar to previous studies, we selected the seasons and meteorological factors related to specific dates during the *in vitro* fertilization (IVF) process as representatives of the overall environment during the treatment period, which may introduce biases into the research findings. Thirdly, factors such as smoking, alcohol consumption, metabolic health, and the presence of diabetes were not considered, potentially affecting the study’s broad applicability. Additionally, a gap remains in our understanding of the underlying mechanisms, which warrants further investigation.

## Conclusion

A summation of data suggests that the autumn season yields a greater number of retrieved oocytes compared to other seasons. The cohort from summer exhibits the lowest rate of miscarriage and the highest live birth rate, contributing to the most favorable pregnancy outcomes. It appears that summer conditions—characterized by high temperature, high humidity, and abundant sunlight—serve as independent positive factors for live birth rates. Therefore, with respect to ART treatments, strategically timing ovulation induction and oocyte retrieval may prove advantageous. Specifically, scheduling oocyte retrieval during the summer months could potentially improve pregnancy outcomes.

## Data Availability

The raw data supporting the conclusions of this article will be made available by the authors, without undue reservation.
